# 44 Higher Out-of-pocket Expenses are Associated with Worse Health-Related Quality of Life in Burn Survivors

**DOI:** 10.1093/jbcr/irad045.018

**Published:** 2023-05-15

**Authors:** Cole Holan, Gretchen Carrougher, Barclay Stewart, Nicole Gibran, Caitlin Orton, Clifford Sheckter

**Affiliations:** University of Texas Austin, Austin, Texas; University of Washington, Seattle, Washington; University of Washington, Seattle, Washington; University of Washington, Seattle, Washington; University of Washington, Seattle, Washington; Stanford University, Palo Alto, California; University of Texas Austin, Austin, Texas; University of Washington, Seattle, Washington; University of Washington, Seattle, Washington; University of Washington, Seattle, Washington; University of Washington, Seattle, Washington; Stanford University, Palo Alto, California; University of Texas Austin, Austin, Texas; University of Washington, Seattle, Washington; University of Washington, Seattle, Washington; University of Washington, Seattle, Washington; University of Washington, Seattle, Washington; Stanford University, Palo Alto, California; University of Texas Austin, Austin, Texas; University of Washington, Seattle, Washington; University of Washington, Seattle, Washington; University of Washington, Seattle, Washington; University of Washington, Seattle, Washington; Stanford University, Palo Alto, California; University of Texas Austin, Austin, Texas; University of Washington, Seattle, Washington; University of Washington, Seattle, Washington; University of Washington, Seattle, Washington; University of Washington, Seattle, Washington; Stanford University, Palo Alto, California; University of Texas Austin, Austin, Texas; University of Washington, Seattle, Washington; University of Washington, Seattle, Washington; University of Washington, Seattle, Washington; University of Washington, Seattle, Washington; Stanford University, Palo Alto, California

## Abstract

**Introduction:**

The care required to recover serious burn injuries is costly. In the US, these costs are often borne by patients. Examining the relationship between out-of-pocket (OOP) costs and health-related quality of life (HRQL) is important to support burn survivors.

**Methods:**

Financial data from a regional burn center were merged with data in the Burn Model System (BMS) National Database. HRQL outcomes included VA-Rand 12 (VR-12) physical component summary (PCS) and mental component summary (MCS) scores. Participant surveys were conducted at 6-, 12-, and 24-months post-injury. VR-12 scores were evaluated using generalized linear models and adjusted for potential confounders (age, sex, insurance/payer, self-identified race/ethnicity, measures of burn injury severity).

**Results:**

644 participants were included, of which 13% (84) had OOP costs. The percentage of participants with OOP costs was 34% for commercial/private, 22% for Medicare, 8% for other, 4% for self-pay, and 0% for workers’ compensation and Medicaid. For participants with OOP expenses, median payments were $875 with an IQR of $368 - 1,728. In addition to markers of burn injury severity, OOP costs were negatively associated with PCS scores at 6-months (coefficient -0.002, p< 0.001) and 12-months post-injury (coefficient -0.001, p=0.004). There were no significant associations with PCS scores at 24 months post-injury or MCS scores at any interval.

**Conclusions:**

Participants with commercial/private or Medicare payer had higher financial liability than other payers. Higher OOP expenses were negatively associated with physical HRQL for at least 12 months after injury.

**Applicability of Research to Practice:**

Financial toxicity is a real phenomenon in burn survivors. Burn care providers should be attuned to their patients' finances and spending and connect them with resources when appropriate.

